# Critical Role of p53 Upregulated Modulator of Apoptosis in Benzyl Isothiocyanate-Induced Apoptotic Cell Death

**DOI:** 10.1371/journal.pone.0032267

**Published:** 2012-02-16

**Authors:** Marie Lue Antony, Su-Hyeong Kim, Shivendra V. Singh

**Affiliations:** Department of Pharmacology & Chemical Biology, and University of Pittsburgh Cancer Institute, University of Pittsburgh School of Medicine, Pittsburgh, Pennsylvania, United States of America; Wayne State University School of Medicine, United States of America

## Abstract

Benzyl isothiocyanate (BITC), a constituent of edible cruciferous vegetables, decreases viability of cancer cells by causing apoptosis but the mechanism of cell death is not fully understood. The present study was undertaken to determine the role of Bcl-2 family proteins in BITC-induced apoptosis using MDA-MB-231 (breast), MCF-7 (breast), and HCT-116 (colon) human cancer cells. The B-cell lymphoma 2 interacting mediator of cell death (Bim) protein was dispensable for proapoptotic response to BITC in MCF-7 and MDA-MB-231 cells as judged by RNA interference studies. Instead, the BITC-treated MCF-7 and MDA-MB-231 cells exhibited upregulation of p53 upregulated modulator of apoptosis (PUMA) protein. The BITC-mediated induction of PUMA was relatively more pronounced in MCF-7 cells due to the presence of wild-type p53 compared with MDA-MB-231 with mutant p53. The BITC-induced apoptosis was partially but significantly attenuated by RNA interference of PUMA in MCF-7 cells. The PUMA knockout variant of HCT-116 cells exhibited significant resistance towards BITC-induced apoptosis compared with wild-type HCT-116 cells. Attenuation of BITC-induced apoptosis in PUMA knockout HCT-116 cells was accompanied by enhanced G2/M phase cell cycle arrest due to induction of p21 and down regulation of cyclin-dependent kinase 1 protein. The BITC treatment caused a decrease in protein levels of Bcl-xL (MCF-7 and MDA-MB-231 cells) and Bcl-2 (MCF-7 cells). Ectopic expression of Bcl-xL in MCF-7 and MDA-MB-231 cells and that of Bcl-2 in MCF-7 cells conferred protection against proapoptotic response to BITC. Interestingly, the BITC-treated MDA-MB-231 cells exhibited induction of Bcl-2 protein expression, and RNA interference of Bcl-2 in this cell line resulted in augmentation of BITC-induced apoptosis. The BITC-mediated inhibition of MDA-MB-231 xenograft growth *in vivo* was associated with the induction of PUMA protein in the tumor. In conclusion, the results of the present study indicate that Bim-independent apoptosis by BITC in cancer cells is mediated by PUMA.

## Introduction

Bioactive compounds from dietary sources continue to draw attention for possible use to prevent breast cancer [1]–[3], which is a leading cause of cancer-related mortality in American women [4]. Cruciferous vegetable constituent benzyl isothiocyanate (BITC) is one such compound with compelling preclinical evidence for preventive efficacy against breast cancer in experimental rodents. Mammary cancer prevention using BITC was first demonstrated by Wattenberg in a rat model of chemically-induced cancer [5]. BITC administration prior to the carcinogen challenge inhibited 7,12-dimethylbenz[a]anthracene-induced mammary tumor development in female Sprague-Dawley rats [5]. Studies from our laboratory have revealed that BITC administration in the diet confers significant protection against mammary cancer development in MMTV-*neu* transgenic mice [6]. The BITC-mediated inhibition of breast cancer xenograft growth *in vivo* has also been documented [7], [8].

We have shown previously that BITC-mediated prevention of mammary cancer development in MMTV-*neu* mice is associated with inhibition of cell proliferation and increased apoptosis [6]. In agreement with our findings [6], BITC-mediated inhibition of 4T1 murine breast cancer xenograft growth in BALB/c mice was accompanied by increased apoptosis [8]. In cellular models of human breast cancer (MDA-MB-231 and MCF-7), BITC treatment causes G2/M phase cell cycle arrest and apoptosis induction [9]–[12]. A spontaneously immortalized and non-tumorigenic human mammary epithelial cell line (MCF-10A), originally isolated from a fibrocystic breast disease, is significantly more resistant to BITC-induced apoptosis compared with breast cancer cells [11]. The mechanism by which BITC causes cell death is not fully understood, but proapoptotic response to this agent in human breast cancer cells is intimately linked to production of reactive oxygen species (ROS) because of inhibition of complex III of the mitochondrial respiratory chain [12]. Activation of caspases and suppression of X-linked inhibitor of apoptosis protein are other mechanistic events associated with BITC-induced apoptosis in breast cancer cells [11]–[13]. We have also observed other novel pharmacological responses for BITC, including inhibition of oncogenic actions of leptin and suppression of epithelial-mesenchymal transition [14], [15].

Mitochondria-mediated apoptosis downstream of ROS production and upstream of caspase activation is regulated by Bcl-2 family proteins, which function to either inhibit (*e.g.*, Bcl-2, Bcl-xL etc.) or facilitate (*e.g.*, Bak, Bax, and Bim) apoptosis [16]–[20]. We have already established that SV40 immortalized mouse embryonic fibroblasts derived from Bax and Bak double knockout mice are significantly more resistant to BITC-induced apoptosis compared with those derived from the wild-type mice [11]. However, the role of other members of the Bcl-2 family proteins in regulation of BITC-induced apoptosis remains elusive. The present study logically extends our previous findings [11]–[13] to systematically investigate the role of B-cell lymphoma 2 interacting mediator of cell death (Bim), p53 upregulated modulator of apoptosis (PUMA), Bcl-xL, and Bcl-2 proteins in regulation of BITC-induced apoptosis using MCF-7 (breast), MDA-MB-231 (breast), and HCT-116 (colon) human cancer cells as a model.

## Results

### Bim is dispensable for BITC-induced apoptosis in MCF-7 and MDA-MB-231 cells

The c-Jun N-terminal kinase (JNK) is often implicated in ROS-dependent apoptosis by different stimuli, including certain natural agents [21]–[24]. The JNK activation results in phosphorylation of the BH3-only protein Bim leading to activation of multidomain proapoptotic protein Bax [25], [26]. Our previous studies have indicated that BITC treatment causes ROS/JNK-dependent activation of Bax in breast cancer cells [12]. Thus, it was logical to test whether BITC-induced apoptosis was mediated by Bim. The BITC-induced apoptosis, as judged by analysis of histone-associated DNA fragment release into the cytosol, was not attenuated by RNA interference of Bim in either MCF-7 or MDA-MB-231 cells (results not shown). These results indicated that Bim protein was dispensable for proapoptotic response to BITC at least in MCF-7 and MDA-MB-231 cells.

### BITC treatment increases PUMA protein expression in MCF-7 and MDA-MB-231 cells

**Figure 1:**
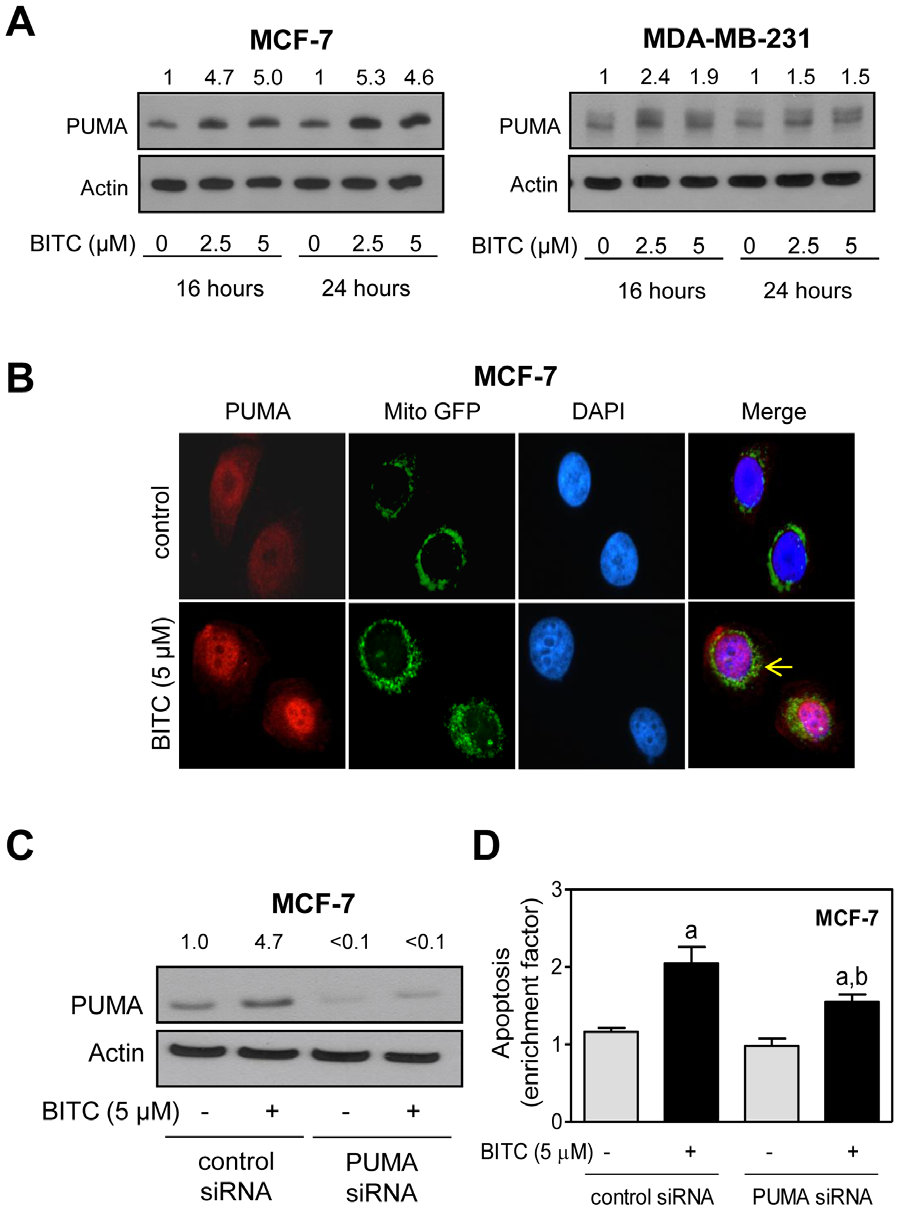
BITC treatment increases PUMA protein level in MCF-7 and MDA-MB-231 cells. (A) Western blotting for PUMA using lysates from MCF-7 and MDA-MB-231 cells treated with DMSO (control) or BITC (2.5 or 5 µM) for the indicated time periods. Number above band indicates change in level compared to corresponding DMSO-treated control. (B) Immunofluorescence microscopy for PUMA in Mito GFP expressing MCF-7 cells after 24 h treatment with DMSO or 5 µM BITC (100× objective magnification). Arrow represents localization of PUMA in mitochondrion. (C) Immunoblotting for PUMA using lysates from MCF-7 cells transiently transfected with a control siRNA or PUMA-targeted siRNA and treated for 24 h with DMSO or 5 µM BITC. (D) Quantitation of histone-associated DNA fragment release into the cytosol (a measure of apoptosis) in MCF-7 cells transiently transfected with a control siRNA or PUMA-targeted siRNA and treated for 24 h with DMSO or 5 µM BITC. Results are expressed as enrichment relative to corresponding DMSO-treated control. Data represent mean ± SD (n = 2–3). Significantly different (P<0.05) compared with ^a^respective DMSO-treated control and ^b^between control siRNA transfected and PUMA siRNA transfected cells by one-way ANOVA followed by Bonferroni's multiple comparison test. The experiments were repeated twice and data from one representative experiment are shown.

PUMA is another BH3-only member of the Bcl-2 family that facilitates apoptosis by different stimuli [20]. For example, PUMA has been shown to indirectly activate Bax in the absence of Bim and Bid [27]. There was a marked increase in the levels of PUMA protein after treatment with BITC in both MCF-7 and MDA-MB-231 cells (Fig. 1A). However, this response was relatively more pronounced in the wild-type p53 expressing MCF-7 cells than in the MDA-MB-231 cells, which express mutant p53 (Fig. 1A). Immunofluorescence microscopy confirmed BITC-mediated induction of PUMA in MCF-7 cells (Fig. 1B). Because BITC-mediated induction of PUMA was relatively more pronounced in MCF-7 cells, we used this cell line for functional assays involving RNA interference. Level of PUMA protein was decreased by >90% upon transient transfection of MCF-7 cells with the PUMA-targeted small interfering RNA (siRNA) in comparison with cells transfected with a control siRNA (Fig. 1C). Furthermore, the BITC-mediated induction of PUMA was fully abolished by its knockdown. Moreover, knockdown of PUMA protein conferred partial but statistically significant protection against BITC-mediated increase in histone-associated DNA fragment release into the cytosol (Fig. 1D). These results indicated that PUMA induction potentially contributed to BITC-induced apoptosis in MCF-7 cells.

**Figure 2:**
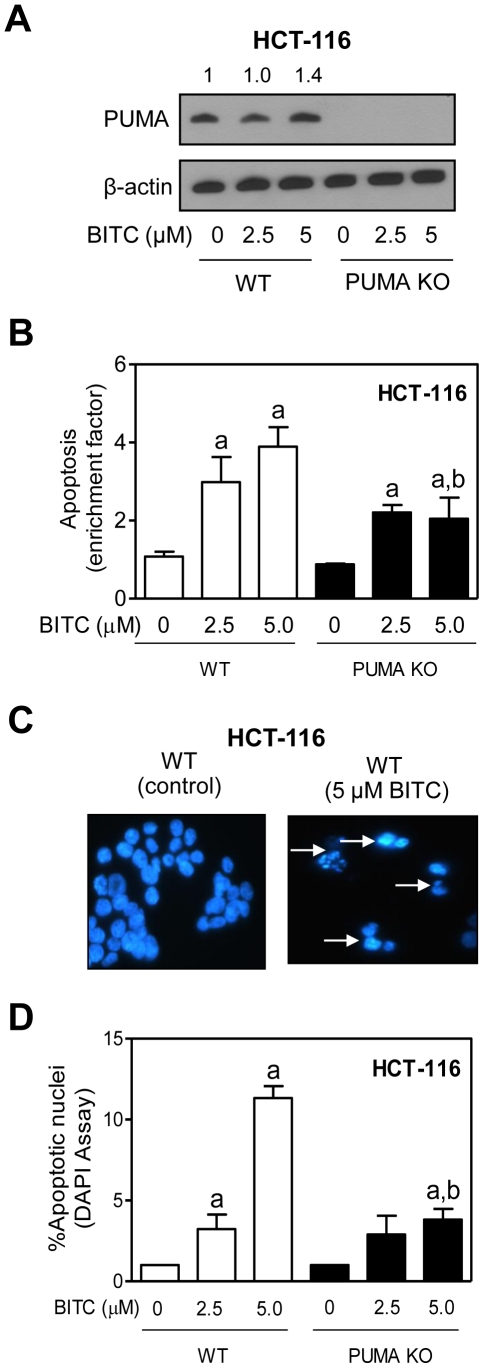
PUMA knockout HCT-116 cells are partially resistant to BITC-induced apoptosis. (A) Immunoblotting for PUMA protein using lysates from wild-type HCT-116 cells (WT) and PUMA knockout HCT-116 cells (PUMA KO) following 24 h treatment with DMSO or BITC (2.5 or 5 µM). (B) Quantitation of histone-associated DNA fragment release into the cytosol in WT and PUMA KO HCT-116 cells after 24 h treatment with DMSO or BITC (2.5 or 5 µM). Results are expressed as enrichment relative to corresponding DMSO-treated control. Data represent mean ± SD (n = 4). Significantly different (P<0.05) compared with ^a^respective DMSO-treated control and ^b^between WT and PUMA KO HCT-116 cells by one-way ANOVA followed by Bonferroni's multiple comparison test. (C) Visualization of apoptotic nuclei (DAPI assay) with condensed and fragmented DNA (identified by arrows) in WT HCT-116 cells after 24-hour treatment with DMSO or 5 µM BITC. (D) Quantitation of apoptotic nuclei in WT and PUMA KO HCT-116 cells following 24 h treatment with DMSO or BITC (2.5 or 5 µM). Data represent mean ± SD (n = 3). Significantly different (P<0.05) compared with ^a^respective DMSO-treated control and ^b^between WT and PUMA KO cells by one-way ANOVA followed by Bonferroni's multiple comparison test. Each experiment was repeated at least twice.

Next, we questioned if PUMA-dependence of BITC-induced apoptosis was unique to the MCF-7 cells. We used wild-type and PUMA knockout variant of HCT-116 cells to address this question. As can be seen in Fig. 2A, level of PUMA protein was modestly increased after 24 h treatment of wild-type HCT-116 cells with 5 µM BITC. The BITC treatment resulted in a dose-dependent and significant increase in histone-associated DNA fragment release into the cytosol over DMSO-treated control in wild-type HCT-116 cells (Fig. 2B). The PUMA knockout HCT-116 cells were relatively more resistant to BITC-induced apoptosis compared with wild-type cells (Fig. 2B). Representative microscopic images depicting apoptotic cells with condensed and fragmented DNA in wild-type HCT-116 cells after 24 h treatment with DMSO (control) and 5 µM BITC are shown in Fig. 2C (apoptotic nuclei are marked with arrows). In agreement with results shown in Fig. 2B, PUMA knockout HCT-116 cells were significantly more resistant to BITC-induced apoptosis in comparison with wild-type HCT-116 cells at least at the 5 µM concentration (Fig. 2D). Collectively, these results indicated that PUMA-dependence of BITC-induced apoptosis was not a cell line-specific response.

### PUMA deficiency increases BITC-mediated G2/M phase cell cycle arrest in HCT-116 cells

**Figure 3:**
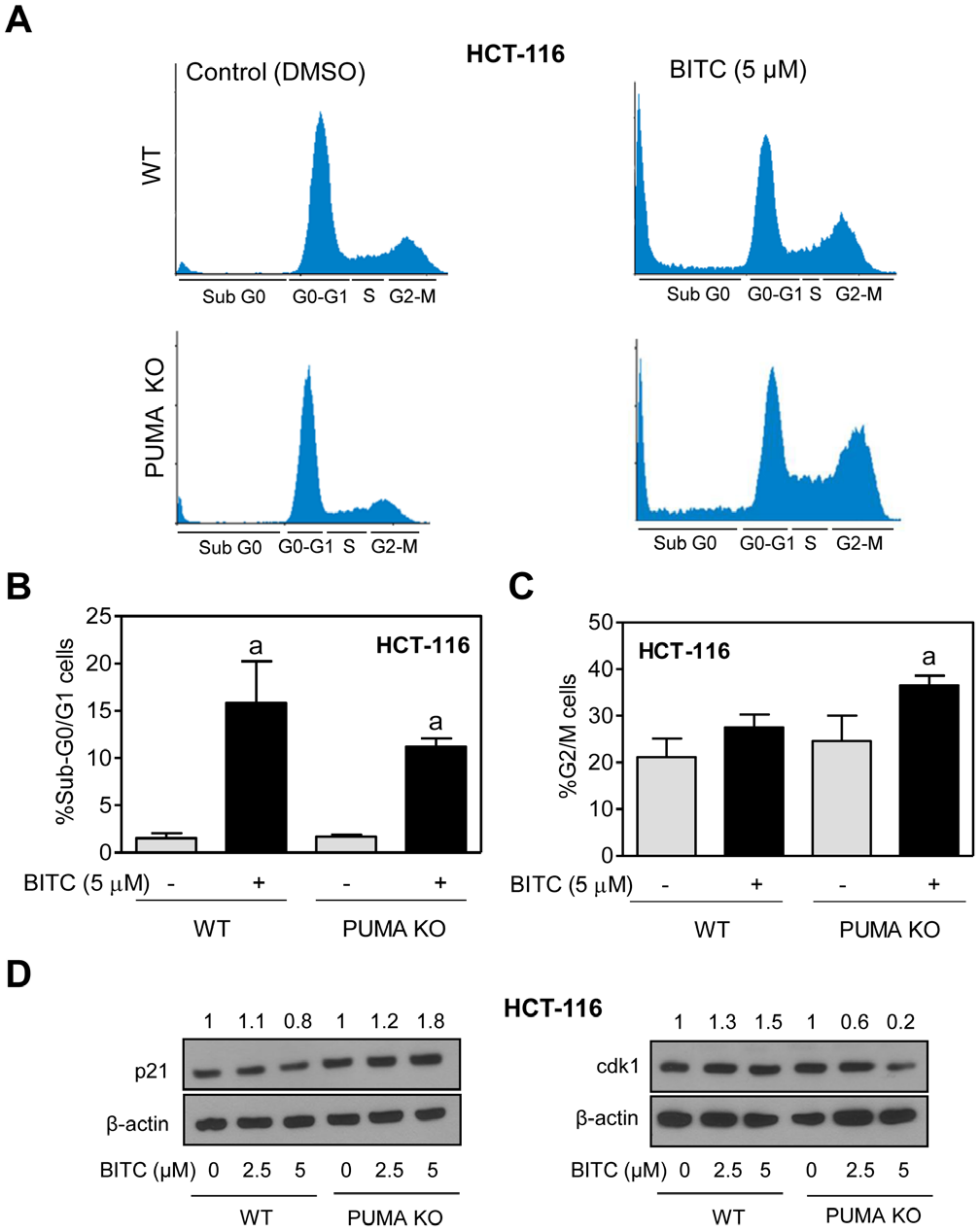
BITC treatment causes G2/M phase cell cycle arrest in PUMA knockout HCT-116 cells. (A) Representative flow histograms depicting cell cycle distribution in wild-type HCT-116 cells (WT) and PUMA knockout HCT-116 cells (PUMA KO) after 24 h treatment with DMSO or 5 µM BITC. Quantitation of (B) sub-G0/G1 and (C) G2/M fraction in WT and PUMA KO HCT-116 cells after 24 h treatment with DMSO or 5 µM BITC. The experiment was repeated twice and merged data from both the experiments are shown. Data represent mean ± SD (n = 4). ^a^Significantly different (P<0.05) compared with DMSO-treated control by one-way ANOVA followed by Bonferroni's multiple comparison test. (D) Immunoblotting for p21 and cdk1 using lysates from WT and PUMA KO HCT-116 cells treated for 24 h with DMSO or the indicated concentrations of BITC. Each experiment was repeated at least twice.

We have shown previously that BITC treatment causes G2/M phase cell cycle arrest in breast cancer cells [11]. We designed experiments using wild-type and PUMA knockout HCT-116 cells to determine if PUMA deficiency affected BITC-mediated cell cycle arrest. Fig. 3A shows representative flow histograms for cell cycle distribution in wild-type and PUMA knockout HCT-116 cells after 24 h treatment with DMSO (control) or 5 µM BITC. As can be seen in Fig. 3B, BITC treatment resulted in a significant increase in fraction of sub-diploid (apoptotic) cells in both wild-type and PUMA knockout HCT-116 cells. Consistent with data shown in Fig. 2 (B,D), the BITC-mediated enrichment of sub-diploid fraction was relatively more pronounced in the wild-type HCT-116 cells than in its PUMA knockout variant (Fig. 3B). Furthermore, the BITC-induced G2/M phase cell cycle arrest was relatively more pronounced in the PUMA knockout cells compared with wild-type HCT-116 cells (Fig. 3C). The BITC-treated PUMA knockout cells exhibited an increase in protein level of p21, but this effect was not evident in the wild-type HCT-116 cells (Fig. 3D). Finally, BITC treatment caused a modest increase in protein level of cyclin-dependent kinase 1 (cdk1) (30–50% increase over DMSO-treated control) in wild-type HCT-116 cells (Fig. 3D). In contrast, expression of cdk1 protein was decreased markedly upon treatment of PUMA knockout HCT-116 cells with BITC (Fig. 3D). These results indicated that attenuation of BITC-induced apoptosis in PUMA knockout HCT-116 cells was accompanied by an increase in G2/M phase cell cycle arrest due to induction of p21 and downregulation of cdk1 protein.

### BITC treatment decreases levels of Bcl-xL and Bcl-2 proteins in MCF-7 cells

**Figure 4:**
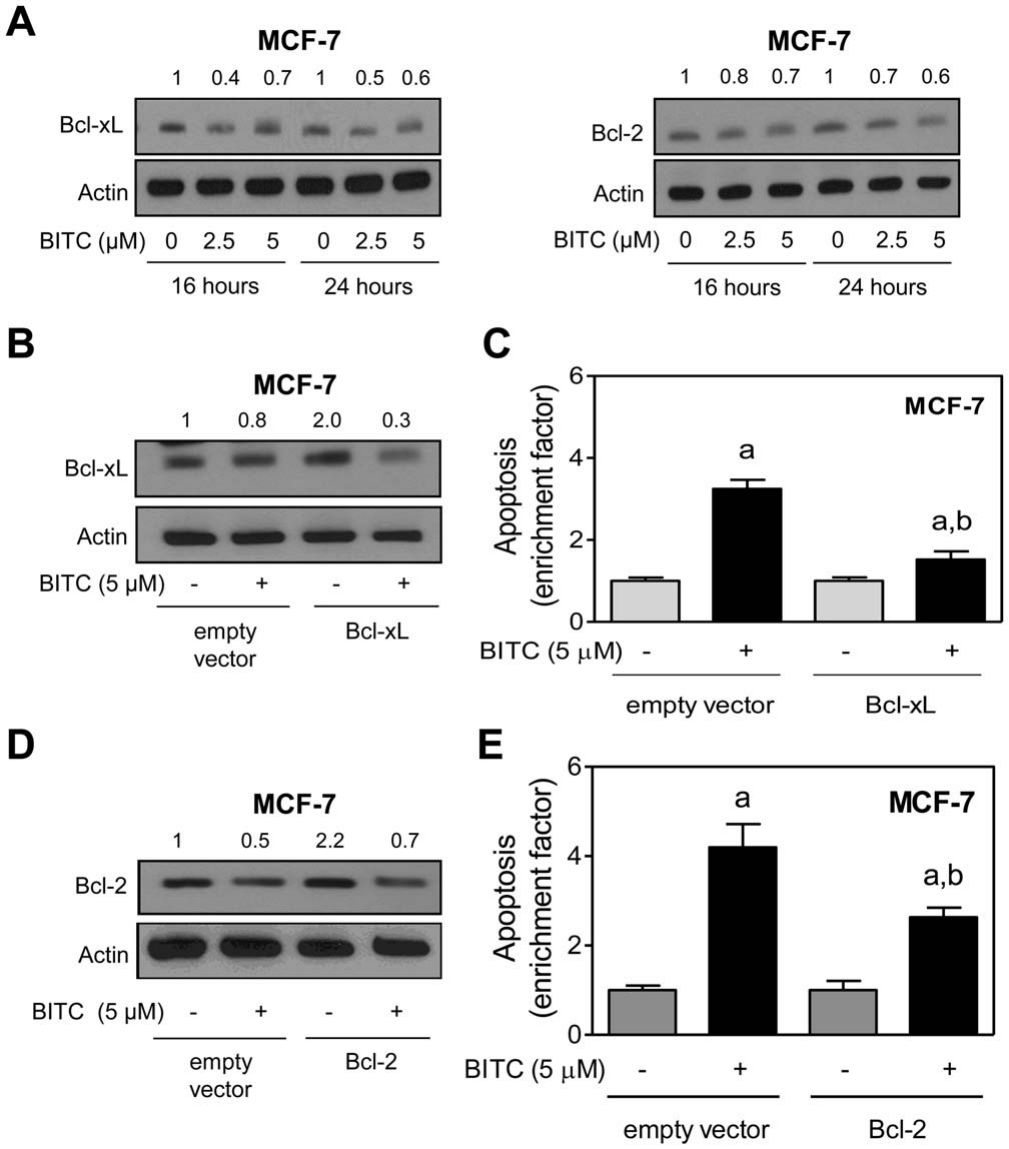
Overexpression of Bcl-xL and Bcl-2 confers protection against BITC-induced apoptosis in MCF-7 cells. (A) Western blotting for Bcl-xL and Bcl-2 using lysates from MCF-7 cells treated with DMSO (control) or BITC (2.5 or 5 µM) for the indicated time periods. Number above band indicates change in level compared to the corresponding DMSO-treated control. (B) Western blotting for Bcl-xL using lysates from MCF-7 cells transiently transfected with empty vector or Bcl-xL plasmid and treated for 24 h with DMSO or 5 µM BITC. Number above band represents change in level relative to empty vector transfected cells treated with DMSO (first lane). (C) Quantitation of histone-associated DNA fragment release into the cytosol in MCF-7 cells transiently transfected with empty vector or vector encoding for Bcl-xL and treated for 24 h with DMSO or 5 µM BITC. (D) Western blotting for Bcl-2 using lysates from MCF-7 cells transiently transfected with empty vector or Bcl-2 plasmid and treated for 24 h with DMSO or 5 µM BITC. Number above band represents change in level relative to empty vector transfected cells treated with DMSO (first lane). (E) Quantitation of histone-associated DNA fragment release into the cytosol in MCF-7 cells transiently transfected with empty vector or vector encoding for Bcl-2 and treated for 24 h with DMSO or 5 µM BITC. Data in C, E are expressed as enrichment relative to corresponding DMSO-treated control (mean ± SD, n = 3). Significantly different (P<0.05) ^a^compared with corresponding DMSO-treated control, and ^b^between empty vector transfected cells and Bcl-xL or Bcl-2 overexpressing cells by one-way ANOVA followed by Bonferroni's multiple comparison test. The experiments were repeated twice and data from one representative experiment are shown.

The PUMA protein serves to activate Bax by relieving inhibition by the anti-apoptotic Bcl-2 family members, including Bcl-2, Bcl-xL, and Mcl-1 [20]. We have shown previously that BITC-induced apoptosis in MCF-7 and MDA-MB-231 cells is associated with suppression of Bcl-xL and/or Bcl-2 [11]. However, functional studies to test the role of these proteins in the context of BITC-induced apoptosis in breast cancer cells are lacking. As shown in Fig. 4A, BITC treatment resulted in suppression of both Bcl-xL and Bcl-2 protein levels in MCF-7 cells. Ectopic expression of Bcl-xL through transient transfection in MCF-7 cells (Fig. 4B) conferred partial but statistically significant protection against BITC-induced apoptosis (Fig. 4C). Similarly, Bcl-2 overexpressing MCF-7 cells (Fig. 4D) were significantly more resistant to BITC-mediated enrichment of histone-associated DNA fragment release into the cytosol compared with cells transiently transfected with the empty vector (Fig. 4E). These results indicated that BITC-induced apoptosis in MCF-7 cells was caused by downregulation of both Bcl-xL and Bcl-2 proteins.

### Opposing effect of BITC on Bcl-xL and Bcl-2 protein levels in MDA-MB-231 cells

**Figure 5:**
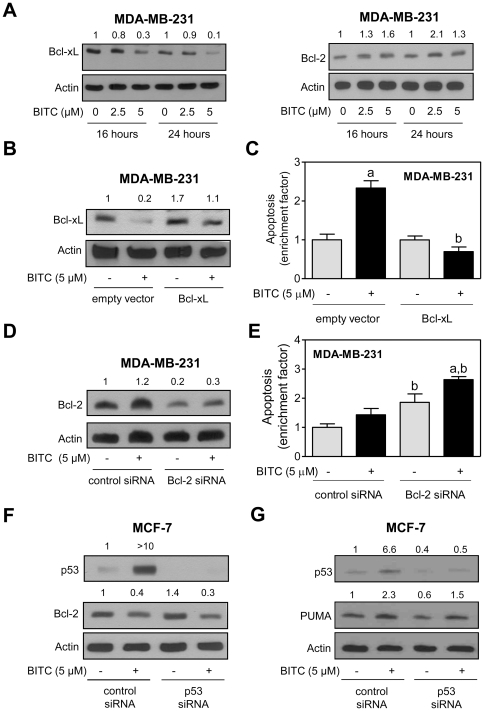
Opposing effect of BITC treatment on levels of Bcl-xL and Bcl-2 proteins in MDA-MB-231 cells. (A) Western blotting for Bcl-xL and Bcl-2 using lysates from MDA-MB-231 cells treated with DMSO (control) or BITC (2.5 or 5 µM) for the indicated time periods. Number above band indicates change in level compared to the corresponding DMSO-treated control. (B) Western blotting for Bcl-xL and (C) quantitation of histone-associated DNA fragment release into the cytosol in MDA-MB-231 cells transiently transfected with empty vector or vector encoding for Bcl-xL and treated for 24 h with DMSO or 5 µM BITC. Results are expressed as enrichment relative to corresponding DMSO-treated control. Data represent mean ± SD (n = 3). Significantly different (P<0.05) ^a^compared with corresponding DMSO-treated control, and ^b^between empty vector transfected cells and Bcl-xL overexpressing cells by one-way ANOVA followed by Bonferroni's multiple comparison test. (D) Western blotting for Bcl-2 and (E) quantitation of histone-associated DNA fragment release into the cytosol in MDA-MB-231 cells transiently transfected with a control siRNA or the Bcl-2-targeted siRNA and treated for 24 h with DMSO or 5 µM BITC. In panel E, results shown are relative to control siRNA transfected cells treated with DMSO. Data represent mean ± SD (n = 3). Significantly different (P<0.05) ^a^compared with corresponding DMSO-treated control, and ^b^between control siRNA transfected cells and Bcl-2 siRNA transfected cells by one-way ANOVA followed by Bonferroni's multiple comparison test. (F) Western blotting for p53 and Bcl-2 using lysates from MCF-7 cells transiently transfected with a control siRNA or the p53-targeted siRNA and treated for 24 h with DMSO or 5 µM BITC. (G) Western blotting for p53 and PUMA using lysates from MCF-7 cells transiently transfected with a control siRNA or the p53-targeted siRNA and treated for 24 h with DMSO or 5 µM BITC. Number above the bands in 5F, G represents change in level relative to control siRNA transfected cells treated with DMSO. All the experiments were repeated at least twice and representative data from one such experiment are shown.

Similar to MCF-7 cells (Fig. 4A), BITC treatment caused a decrease in protein level of Bcl-xL in MDA-MB-231 cells especially at the 5 µM concentration (Fig. 5A). To the contrary, BITC-treated MDA-MB-231 cells exhibited a marked increase in level of Bcl-2 protein (Fig. 5A). The BITC treatment downregulated Bcl-xL protein expression in MDA-MB-231 cells transiently transfected with both the empty vector and vector encoding for Bcl-xL (Fig. 5B). In agreement with results in MCF-7 cells (Fig. 4C), overexpression of Bcl-xL protein was protective against BITC-mediated apoptosis in MDA-MB-231 cells (Fig. 5C). Because the expression of Bcl-2 protein was increased after BITC treatment in MDA-MB-231 cells, we determined the effect of its knockdown on BITC-induced apoptosis. Level of Bcl-2 protein was decreased by 80% in MDA-MB-231 cells transfected with the Bcl-2-targeted siRNA compared with control siRNA transfected cells (Fig. 5D). Knockdown of Bcl-2 alone increased apoptosis in MDA-MB-231 cells (Fig. 5E). In addition, the BITC-induced apoptosis was increased significantly in MDA-MB-231 cells upon knockdown of Bcl-2 when compared with cells transfected with the control siRNA (Fig. 5E). Collectively, these results indicated that MCF-7 and MDA-MB-231 cells responded differentially to BITC-mediated alterations in Bcl-2 protein expression.

### p53 is dispensable for BITC-mediated downregulation of Bcl-2 protein in MCF-7 cells

Next, we questioned whether differential behavior of MCF-7 *versus* MDA-MB-231 cells to BITC-mediated change in Bcl-2 protein expression was related to difference in p53 status. Twenty-four hour treatment of control siRNA transfected MCF-7 cells to 5 µM BITC resulted in >10-fold increase in level of p53 protein (Fig. 5F). This effect was not observed in MCF-7 cells transfected with the p53-targeted siRNA. However, the BITC-mediated suppression of Bcl-2 protein level was observed in MCF-7 cells transfected with both control siRNA and p53-targetd siRNA (Fig. 5F). These results indicated that BITC-mediated downregulation of Bcl-2 was not influenced by the p53 status at least in MCF-7 cells.

### Role of p53 in BITC-mediated induction of PUMA

We sought to determine the role of p53 in BITC-mediated induction of PUMA using MCF-7 cells. As can be seen in Fig. 5G, MCF-7 cells transfected with the p53-targeted siRNA showed a 60% decrease in the level of p53 protein in comparison with cells transfected with the control siRNA. In addition, the BITC-mediated induction of PUMA was partially reversed upon RNA interference of p53. Two possibilities exist to explain these results: (a) the BITC-mediated induction of PUMA in MCF-7 cells is only partially regulated by p53, and (b) PUMA induction after treatment with BITC in MCF-7 cells transfected with the p53-targeted siRNA is simply a consequence of incomplete knockdown of the p53 protein. Nevertheless, based on data in MDA-MB-231 cells, it is likely that both p53-dependent and -independent mechanisms are responsible for BITC-mediated induction of PUMA at least in breast cancer cells.

### BITC administration causes *in vivo* induction of PUMA in MDA-MB-231 xenografts

**Figure 6:**
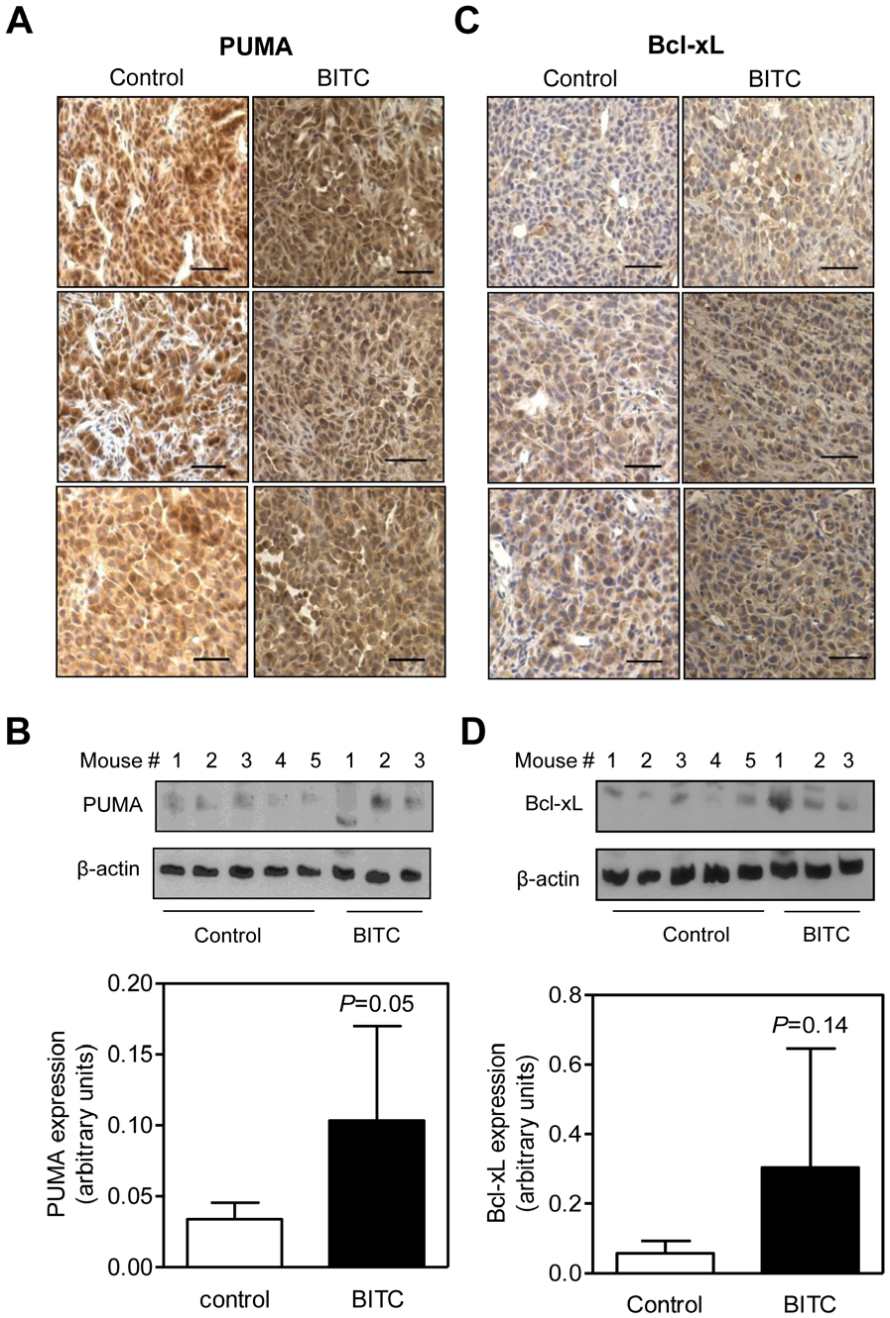
BITC administration increase PUMA expression *in vivo* in MDA-MB-231 xenografts. Immunohistochemical analysis for (A) PUMA and (C) Bcl-xL in MDA-MB-231 tumor sections from control and BITC-treated mice (7). Magnification- 200×; scale bar- 40 µm. Immunoblotting for (B) PUMA and (D) Bcl-xL using tumor supernatants from control and BITC treatment groups. Lower panels in B and D represent densitometric quantitation (arbitrary units). Statistical significance was determined by Student's t-test (n = 5 for control and n = 3 for BITC treatment group).

We used tumor specimens from our previously published MDA-MB-231 xenograft study [7] to determine the *in vivo* effect of BITC administration on the expression of PUMA and Bcl-xL proteins. The MDA-MB-231 tumors from BITC-treated mice exhibited induction of PUMA protein when compared with tumors from vehicle-treated control mice as judged by immunohistochemistry (Fig. 6A) and western blotting (Fig. 6B). Even though BITC treatment resulted in *in vivo* induction of Bcl-xL as well (Fig. 6C), the difference was not significant (Fig. 6D). These results provided *in vivo* evidence for BITC-mediated induction of PUMA protein in MDA-MB-231 xenografts.

## Discussion

The results presented herein indicate that PUMA plays an important role in BITC-induced apoptosis. This conclusion is based on the following observations: (a) the BITC treatment increases the level of PUMA protein in breast cancer cells, which is not a cell line-specific response but relatively more pronounced in cells with wild-type p53 (MCF-7); although RNA interference of p53 alone has minimal impact on BITC-induced apoptosis in MCF-7 cells [13], (b) BITC-induced apoptosis is partially but significantly attenuated by RNA interference of PUMA, (c) the PUMA knockout variant of HCT-116 is significantly more resistant to BITC-induced apoptosis compared with wild-type HCT-116 cells, and (d) BITC administration increases levels of PUMA protein in MDA-MB-231 tumor xenografts *in vivo*. Because BITC-mediated induction of PUMA is discernible in cultured and xenografted breast cancer cells, this protein represents a viable biomarker of BITC response.

Role of p53 in regulation of PUMA expression is well established [20]. PUMA is expressed at a low level in normal tissues but it is highly sensitive to induction in response to a wide variety of stresses [20], [28]. In stressed cells (*e.g.*, DNA damage), p53 is recruited to the two p53-responsive elements in the *PUMA* promoter [29]. Binding of p53 to the *PUMA* promoter alters acetylation of histones H3 and H4 leading to opening of the chromatin structure and hence transcriptional activation [30]. The results of the present study indicate that BITC treatment causes both p53-dependent and p53-independent induction of PUMA as this effect is observed in both MCF-7 and MDA-MB-231 cells. However, as expected the MCF-7 cell line is relatively more sensitive to BITC-mediated induction of PUMA protein compared with the MDA-MB-231 cell line. Further studies are needed to gain insights into the p53-independent mechanism(s) responsible for BITC-mediated induction of PUMA protein especially in the MDA-MB-231 cells. It is possible that this effect in MDA-MB-231 cells is mediated by transcriptional suppression of Slug, which is a negative regulator of PUMA [31]. We have shown previously that BITC-mediated inhibition of epithelial-mesenchymal transition in MDA-MB-231 cells is associated with transcriptional repression of Slug [15]. At the same time, several other transcription factors have been implicated in regulation of *PUMA* expression, including p53 homologue p73 [32], the forkhead family member FOXO3a [33], c-Myc [34], and E2F1 [35], and their involvement in BITC-mediated induction of PUMA protein can't be excluded.

Attenuation of BITC-induced apoptosis in PUMA knockout HCT-116 cells is accompanied by an increase in G2/M phase cell cycle arrest, which is accompanied by induction of p21. These findings are consistent with literature data showing the balance between induction of PUMA and/or suppression of p21 in cells committed for cell cycle arrest or apoptotic death [36]. Conversely, suppression of PUMA [31] or selective induction of cell-cycle regulators [37] is evident in cells resistant to DNA-damage-induced apoptosis.

We have shown previously that ROS generation is a critical event in BITC-induced apoptosis in MDA-MB-231 and MCF-7 cells [12]. Attenuation of apoptosis by ectopic expression of catalase and superoxide dismutase reinforces the notion that ROS provide initial signal for BITC-induced apoptosis at least in breast cancer cells [12]. The BITC-mediated ROS production is associated with inhibition of complex III of the mitochondrial respiratory chain [12]. Notably, PUMA can be induced by oxidative stress in neuronal and colon cancer cells [38], [39]. The PUMA-deficient neurons are resistant to apoptosis induction by oxidative stress [38]. At the same time, Liu et al [40] have shown that PUMA overexpression itself causes ROS generation in colon cancer cells. It would be interesting to determine if ROS function upstream of PUMA induction or PUMA induction is partly responsible for the pro-oxidant effect of BITC in addition to inhibition of mitochondrial electron transport chain. Nevertheless, both ROS production and PUMA induction seem important for BITC-induced apoptosis in breast cancer cells [12, and present study].

The multidomain proapoptotic protein Bax seems necessary for PUMA-mediated apoptosis. For example, the Bax knockout HCT-116 cells are fully resistant to apoptosis induction by PUMA overexpression as well as stimuli leading to PUMA-dependent apoptosis [41]. Moreover, PUMA overexpression has been shown to cause conformational change, polymerization, and mitochondrial translocation of Bax [41], [42]. We have also shown previously that BITC treatment causes ROS-dependent activation (conformational change) and mitochondrial translocation of Bax in MDA-MB-231 cells [12]. Moreover, SV40 immortalized mouse embryonic fibroblasts derived from Bax and Bak double knockout mice are partially but significantly more resistant to BITC-induced apoptosis compared with mouse embryonic fibroblasts derived from wild-type mice [11]. We propose a working mechanistic model involving ROS-dependent induction of PUMA and Bax activation in BITC-induced apoptosis.

PUMA contributes to apoptosis by directly interacting with anti-apoptotic Bcl-2 family members [20]. Direct interaction between anti-apoptotic proteins and PUMA is dependent on its BH3 domain as this interaction is disrupted by deletion or mutations in BH3 domain [28]. The BH3 peptide of PUMA is capable of interacting with anti-apoptotic proteins [43], [44]. Overexpression of PUMA has been shown to cause dissociation of Bax from Bcl-xL [42]. Interestingly, BITC treatment not only causes the induction of PUMA, which is likely to relieve inhibitory effects of Bcl-xL and Bcl-2, but also causes downregulation of Bcl-xL in both MCF-7 and MDA-MB-231 cells and that of Bcl-2 in MCF-7 cells. It is possible that Bcl-xL and Bcl-2 downregulation itself contributes to PUMA-independent apoptosis in our model. However, the molecular basis for differential effect of BITC treatment on the level of Bcl-2 protein in MCF-7 (downregulation) *versus* MDA-MB-231 cells (upregulation) remains elusive. We have already ruled out involvement of p53 in this differential response.

In summary, the present study indicates that: (a) the Bim protein is dispensable for proapoptotic response to BITC in breast cancer cells; (b) PUMA induction and downregulation of Bcl-xL protein contribute to BITC-induced apoptosis, which is not a cell line-specific phenomenon; (c) BITC treatment differentially affects the levels of Bcl-2 protein in MCF-7 *versus* MDA-MB-231 cells; and (d) PUMA deficiency in HCT-116 human colon cancer cell line increases its sensitivity to BITC-induced G2/M phase cell cycle arrest.

## Methods

### Ethics statement

The MDA-MB-231 tumor xenografts from control and BITC-treated mice archived from our previously published study [7] were used in the present study to determine the effect of BITC administration on expression of PUMA and Bcl-xL proteins. Use of mice was approved by the Institutional Animal Care and Use Committee (protocol number 1004983A-4).

### Reagents

BITC was purchased from LKT Laboratories (St. Paul, MN). Stock solution of BITC was prepared in DMSO and an equal volume of DMSO (<0.05%) was added to the controls. Reagents for cell culture including medium, fetal bovine serum, antibiotics and Alexa Fluor 568-conjugated donkey anti-rabbit antibody were purchased from Invitrogen-Life Technologies (Carlsbad, CA). Antibody against Bcl-2 was from DAKO Cytomation (Carpinteria, CA); antibody against Bim was from Cell Signaling Technology (Beverly, MA); anti-p53 antibody was from Calbiochem-EMD Chemicals (Gibbstown, NJ); anti-actin antibody, 4′,6-diamidino-2-phenylindole (DAPI) and propidium iodide were from Sigma-Aldrich (St. Louis, MO). The siRNA targeted against p53, Bcl-2, Bim, and PUMA, and antibodies against Bcl-xL, PUMA, and cdk1 were purchased from Santa Cruz Biotechnology (Santa Cruz, CA). Anti p21 antibody was from BD Biosciences (San Diego, CA). A nonspecific control siRNA was from Qiagen (Germantown, MD). A kit for quantification of histone-associated DNA fragment release into the cytosol was purchased from Roche Applied Science (Indianapolis, IN).

### Cell lines

MCF-7 and MDA-MB-231 cells were obtained from the American Type Culture collection (Manassas, VA), and maintained as described by us previously [11], [12]. Wild-type HCT-116 human colon cancer cell line and its isogenic PUMA knockout variant were generously provided by Dr. Bert Vogelstein (Johns Hopkins University, Baltimore, MD) and cultured in McCoy's 5A modified medium supplemented with 10% fetal bovine serum and penicillin/streptomycin antibiotic mixture.

### Western blotting

Control and BITC-treated cells and tumor tissues from mice treated with vehicle control and BITC were processed for immunoblotting as described by us previously [45]–[47]. Proteins from cell lysates and tumor supernatants were resolved by sodium-dodecyl sulfate polyacrylamide gel electrophoresis and transferred onto membrane. The membrane was incubated with the desired primary antibody for overnight at 4°C after blocking in 5% non-fat dry milk. Immunoreactive bands were visualized by enhanced chemiluminescence method. Densitometric quantitation was done using UN-SCAN-IT software version 5.1 (Silk Scientific Corporation, Orem, Utah, USA).

### RNA interference

MDA-MB-231 and MCF-7 cells were transfected at ∼50% confluency with 100 nM of target specific siRNA or a control siRNA using OligoFECTAMINE (Invitrogen-Life Technologies). Twenty-four hours post transfection, cells were treated with DMSO (control) or BITC for specified time period. Cells were collected and processed for immunoblotting and measurement for histone-associated DNA fragment release into the cytosol.

### Immunofluorescence microscopy for PUMA expression

MCF-7 stably transfected with Mito-GFP were maintained in media supplemented with 400 µg/mL G418. The cells were plated on cover slips and treated with DMSO (control) or 5 µM BITC for 24 h followed by fixing in paraformaldehyde. Cells were permeabilized with Triton X-100 and incubated with blocking buffer containing bovine serum albumin in phosphate-buffered saline (PBS) for 1 h at room temperature. Cells were then incubated with anti-PUMA antibody in blocking buffer overnight at 4°C. Cells were stained with Alexa Fluor 568-conjugated secondary antibody and DAPI prior to mounting. Immunofluorescence was examined under a Leica DC300F fluorescence microscope.

### DAPI assay

DAPI staining was done to quantify apoptotic cells with condensed and fragmented DNA. The DMSO-treated control and BITC-treated cells were fixed in paraformaldehyde and permeabilized with 0.4% Triton X-100. Cells were then stained with 10 ng/mL DAPI for 5 min at room temperature. Apoptotic cells were counted under a fluorescence microscope.

### Flow cytometry and cell cycle analysis

HCT-116 cells (WT and PUM KO) were treated with BITC or DMSO for 24 h and fixed in 70% ice-cold ethanol. Cells were then treated with 100 mg/mL RNaseA and 50 mg/mL propidium iodide, and subjected to flow cytometry (Coulter Epics XL cytometer). Cell cycle distribution was determined as described by us previously [48].

### Transient transfection

MDA-MB-231 and MCF-7 cells were transiently transfected at ∼50–60% confluency with the empty pSFFV-neo vector or pSFFV vector encoding for Bcl-2 or Bcl-xL using FuGENE6 transfection reagent. Transfected cells were treated with DMSO or BITC and processed for immunoblotting and measurement of apoptosis.

### Immunohistochemistry

Immunohistochemistry for PUMA and Bcl-xL in tumor sections was performed as described by us previously for other proteins [47]. Because the expression of PUMA and Bcl-xL was quite robust even in control tumors, quantitation of expression was not performed.

## References

[1] Sahin K, Tuzcu M, Sahin N, Akdemir F, Ozercan I (2011). Inhibitory effects of combination of lycopene and genistein on 7,12- dimethyl benz(a)anthracene-induced breast cancer in rats.. Nutr Cancer.

[2] Sakata M, Ikeda T, Imoto S, Jinno H, Kitagawa Y (2011). Prevention of mammary carcinogenesis in C3H/OuJ mice by green tea and tamoxifen.. Asian Pac J Cancer Prev.

[3] Park K, Choi K, Kim H, Kim K, Lee MH (2009). Isoflavone-deprived soy peptide suppresses mammary tumorigenesis by inducing apoptosis.. Exp Mol Med.

[4] Jemal A, Siegel R, Xu J, Ward E (2010). Cancer Statistics, 2010.. CA Cancer J Clin.

[5] Wattenberg LW (1977). Inhibition of carcinogenic effects of polycyclic hydrocarbons by benzyl isothiocyanate and related compounds.. J Natl Cancer Inst.

[6] Warin R, Chambers WH, Potter DM, Singh SV (2009). Prevention of mammary carcinogenesis in MMTV-*neu* mice by cruciferous vegetable constituent benzyl isothiocyanate.. Cancer Res.

[7] Warin R, Xiao D, Arlotti JA, Bommareddy A, Singh SV (2010). Inhibition of human breast cancer xenograft growth by cruciferous vegetable constituent benzyl isothiocyanate.. Mol Carcinogenesis.

[8] Kim EJ, Hong JE, Eom SJ, Lee JY, Park JH (2011). Oral administration of benzyl-isothiocyanate inhibits solid tumor growth and lung metastasis of 4T1 murine mammary carcinoma cells in BALB/c mice.. Breast Cancer Res Treat.

[9] Zhang Y, Tang L, Gonzalez V (2003). Selected isothiocyanates rapidly induce growth inhibition of cancer cells.. Mol Cancer Ther.

[10] Tseng E, Scott-Ramsay EA, Morris ME (2004). Dietary organic isothiocyanates are cytotoxic in human breast cancer MCF-7 and mammary epithelial MCF-12A cell lines.. Exp Biol Med.

[11] Xiao D, Vogel V, Singh SV (2006). Benzyl isothiocyanate-induced apoptosis in human breast cancer cells is initiated by reactive oxygen species and regulated by Bax and Bak.. Mol Cancer Ther.

[12] Xiao D, Powolny AA, Singh SV (2008). Benzyl isothiocyanate targets mitochondrial respiratory chain to trigger reactive oxygen species-dependent apoptosis in human breast cancer cells.. J Biol Chem.

[13] Kim SH, Singh SV (2010). p53-Independent apoptosis by benzyl isothiocyanate in human breast cancer cells is mediated by suppression of XIAP expression.. Cancer Prev Res.

[14] Kim SH, Nagalingam A, Saxena NK, Singh SV, Sharma D (2011). Benzyl isothiocyanate inhibits oncogenic actions of leptin in human breast cancer cells by suppressing activation of signal transducer and activator of transcription 3.. Carcinogenesis.

[15] Sehrawat A, Singh SV (2011). Benzyl isothiocyanate inhibits epithelial-mesenchymal transition in cultured and xenografted human breast cancer cells.. Cancer Prev Res.

[16] Chao DT, Korsmeyer SJ (1998). Bcl-2 family: regulators of cell death.. Annu Rev Immunol.

[17] Adams JM, Cory S (2007). The Bcl-2 apoptotic switch in cancer development and therapy.. Oncogene.

[18] Akiyama T, Dass CR, Choong PF (2009). Bim-targeted cancer therapy: a link between drug action and underlying molecular changes.. Mol Cancer Ther.

[19] Gillings AS, Balmanno K, Wiggins CM, Johnson M, Cook SJ (2009). Apoptosis and autophagy: BIM as a mediator of tumour cell death in response to oncogene-targeted therapeutics.. FEBS J.

[20] Yu J, Zhang L (2008). PUMA, a potent killer with or without p53.. Oncogene.

[21] Shanlou Q, Keiko M, Qinghong Z, Baoling W, Hisao S (2011). Mimosine-induced apoptosis in C6 glioma cells requires the release of mitochondria-derived reactive oxygen species and p38, JNK activation.. Neurochem Res.

[22] Dhansekaran DN, Reddy EP (2008). JNK signaling and apoptosis.. Oncogene.

[23] Xiao D, Choi S, Johnson DE, Vogel VG, Johnson CS (2004). Diallyl trisulfide-induced apoptosis in human prostate cancer cells involves c-Jun N-terminal kinase and extracellular-signal regulated kinase-mediated phosphorylation of Bcl-2.. Oncogene.

[24] Xiao D, Zeng Y, Prakash L, Badmaev V, Majeed M (2011). Reactive oxygen species-dependent apoptosis by gugulipid extract of *Ayurvedic* medicine plant *Commiphora mukul* in human prostate cancer cells is regulated by c-JUN N-terminal kinase.. Mol Pharmacol.

[25] Putcha GV, Le S, Frank S, Besirli CG, Clark K (2003). JNK-mediated BIM phosphorylation potentiates BAX-dependent apoptosis.. Neuron.

[26] Lei K, Davis RJ (2003). JNK phosphorylation of Bim-related members of the Bcl2 family induces Bax-dependent apoptosis.. Proc Natl Acad Sci USA.

[27] Jabbour AM, Heraud JE, Daunt CP, Kaufmann T, Sandow J (2009). Puma indirectly activates Bax to cause apoptosis in the absence of Bid or Bim.. Cell Death Differ.

[28] Yu J, Zhang L, Hwang PM, Kinzler KW, Vogelstein B (2001). PUMA induces the rapid apoptosis of colorectal cancer cells.. Mol Cell.

[29] Kaeser MD, Iggo RD (2002). Chromatin immunoprecipitation analysis fails to support the latency model for regulation of p53 DNA binding activity *in vivo*.. Proc Natl Acad Sci USA.

[30] Wang P, Yu J, Zhang L (2007). The nuclear function of p53 is required for PUMA-mediated apoptosis induced by DNA damage.. Proc Natl Acad Sci USA.

[31] Wu WS, Heinrichs S, Xu D, Garrison SP, Zambetti GP (2005). Slug antagonizes p53-mediated apoptosis of hematopoietic progenitors by repressing puma.. Cell.

[32] Melino G, Bernassola F, Ranalli M, Yee K, Zong WX (2004). p73 Induces apoptosis via PUMA transactivation and Bax mitochondrial translocation.. J Biol Chem.

[33] You H, Pellegrini M, Tsuchihara K, Yamamoto K, Hacker G (2006). FOXO3a-dependent regulation of Puma in response to cytokine/growth factor withdrawal.. J Exp Med.

[34] Fernandez PC, Frank SR, Wang L, Schroeder M, Liu S (2003). Genomic targets of the human c-Myc protein.. Genes Dev.

[35] Hershko T, Ginsberg D (2004). Up-regulation of Bcl-2 homology 3 (BH3)-only proteins by E2F1 mediates apoptosis.. J Biol Chem.

[36] Iyer NG, Chin SF, Ozdag H, Daigo Y, Hu DE (2004). p300 regulates p53-dependent apoptosis after DNA damage in colorectal cancer cells by modulation of PUMA/p21 levels.. Proc Natl Acad Sci USA.

[37] Jackson JG, Pereira-Smith OM (2006). p53 is preferentially recruited to the promoters of growth arrest genes *p21* and *GADD45* during replicative senescence of normal human fibroblasts.. Cancer Res.

[38] Steckley D, Karajgikar M, Dale LB, Fuerth B, Swan P (2007). Puma is a dominant regulator of oxidative stress induced Bax activation and neuronal apoptosis.. J Neurosci.

[39] Macip S, Igarashi M, Berggren P, Yu J, Lee SW (2003). Influence of induced reactive oxygen species in p53-mediated cell fate decisions.. Mol Cell Biol.

[40] Liu Z, Lu H, Shi H, Du Y, Yu J (2005). PUMA overexpression induces reactive oxygen species generation and proteasome-mediated stathmin degradation in colorectal cancer cells.. Cancer Res.

[41] Yu J, Wang Z, Kinzler KW, Vogelstein B, Zhang L (2003). PUMA mediates the apoptotic response to p53 in colorectal cancer cells.. Proc Natl Acad Sci USA.

[42] Ming L, Wang P, Bank A, Yu J, Zhang L (2006). PUMA dissociates Bax and Bcl-X(L) to induce apoptosis in colon cancer cells.. J Biol Chem.

[43] Chen L, Willis SN, Wei A, Smith BJ, Fletcher JI (2005). Differential targeting of prosurvival Bcl-2 proteins by their BH3-only ligands allows complementary apoptotic function.. Mol Cell.

[44] Kuwana T, Bouchier-Hayes L, Chipuk JE, Bonzon C, Sullivan BA (2005). BH3 domains of BH3-only proteins differentially regulate Bax-mediated mitochondrial membrane permeabilization both directly and indirectly.. Mol Cell.

[45] Xiao D, Srivastava SK, Lew KL, Zeng Y, Hershberger P (2003). Allyl isothiocyanate, a constituent of cruciferous vegetables, inhibits proliferation of human prostate cancer cells by causing G_2_/M arrest and inducing apoptosis.. Carcinogenesis.

[46] Xiao D, Lew KL, Kim YA, Zeng Y, Hahm ER (2006). Diallyl trisulfide suppresses growth of PC-3 human prostate cancer xenograft *in vivo* in association with Bax and Bak induction.. Clin Cancer Res.

[47] Powolny AA, Bommareddy A, Hahm ER, Normolle DP, Beumer JH (2011). Chemopreventative potential of the cruciferous vegetable constituent phenethyl isothiocyanate in a mouse model of prostate cancer.. J Natl Cancer Inst.

[48] Herman-Antosiewicz A, Singh SV (2005). Checkpoint kinase 1 regulates diallyl trisulfide-induced mitotic arrest in human prostate cancer cells.. J Biol Chem.

